# Prognostic value of preoperative sarcopenia and inflammatory markers in patients undergoing pancreaticoduodenectomy

**DOI:** 10.3389/fsurg.2026.1855285

**Published:** 2026-08-12

**Authors:** Hang Yu, JianJun Wu, XiuLin Wang, JiaHao Luo, WeiLong Du, YuXue Luo, ZhongPing Xu, YunBing Wang, Xiong Ding

**Affiliations:** 1 Department of Hepatobiliary Surgery, The Second Affiliated Hospital of Chongqing Medical University, Chongqing, China; 2 Department of General Surgery, Dushan County People's Hospital, Dushan County, Qiannan Buyei and Miao Autonomous Prefecture, Guizhou Province, China; 3 The Second Clinical College, Chongqing Medical University, Chongqing, China; 4 Department of Neurosurgery, The Second Affiliated Hospital of Chongqing Medical University, Chongqing, China; 5 Clinical Nutrition Department, Chongqing University FuLing Hospital, Chongqing, China

**Keywords:** complications, inflammatory markers, overall surviva, pancreaticoduodenectomy, sarcopenia

## Abstract

**Introduction:**

The combined role of preoperative sarcopenia and inflammatory response in patients undergoing pancreaticoduodenectomy (PD) remains unclear. This study investigates the predictive value of sarcopenia combined with inflammatory markers for postoperative overall survival (OS).

**Methods:**

We retrospectively analyzed 318 patients who underwent PD between February 2019 and March 2025. Sarcopenia was defined by CT-derived skeletal muscle index (SMI) using sex- and BMI-specific thresholds. Inflammatory markers included NLR, PLR, LMR and CAR. Optimal cutoffs were determined via ROC curves, and prognostic factors were identified using Cox proportional hazards models. Survival analysis was performed using the Kaplan–Meier method.

**Results:**

Of the 318 patients, 196 (61.64%) were diagnosed with preoperative sarcopenia. Multivariable analysis showed no significant association between sarcopenia or inflammatory markers and postoperative complications evaluated in this study (*P* > 0.05). CA19-9 (OR:0.999,P=0.027) was a protective factor for POPF. SMI (OR: 1.165, *P* = 0.041), neutrophils (OR: 1.191, *P* = 0.005), and Operative blood loss (OR: 1.001, *P* = 0.022) were independent risk factors for 30-day readmission. Multivariable analysis identified sarcopenia (HR=1.935,P=0.012) as an independent predictor of OS. Based on ROC curve analysis, the optimal cutoff values for NLR and CAR were exploratorily identified as 2.705 and 0.372. Survival analysis revealed that patients with sarcopenia combined with either high NLR (>2.705) or high CAR (>0.372) had significantly shorter mean OS compared to non-sarcopenic patients with low NLR or low CAR (P=0.007,P=0.001). Specifically, the concurrent presence of sarcopenia and high NLR was associated with the highest mortality risk (HR=5.155,P=0.007), followed by the combination of sarcopenia and high CAR (HR=2.808,P=0.001).

**Conclusion:**

Preoperative sarcopenia is an independent predictor of poor OS following PD. The coexistence of sarcopenia and a high inflammatory state (i.e., high NLR or high CAR) significantly increases the risk of mortality. Therefore, preoperative assessment of skeletal muscle mass and inflammatory status may be crucial for identifying high-risk patients.

## 1 Introduction

Pancreaticoduodenectomy (PD) is an effective curative procedure for benign and malignant tumors of the ampulla of Vater, the head of pancreas, and the duodenum; however, it remains one of the most challenging procedures in abdominal surgery (1). Despite advancements in surgical techniques and perioperative management that have reduced the mortality rate associated with PD to approximately 3% (2, 3), PD necessitates extensive resection and complex anastomotic reconstruction, resulting in a postoperative complication rate exceeding 50% (4, 5). These complications not only prolong patients’ hospital stays and increase medical costs, but also compromise their overall survival (OS) (6, 7).

Therefore, identifying risk factors associated with postoperative complications and OS is crucial for improving the prognosis of patients undergoing PD. Sarcopenia is a syndrome characterized by a progressive decline in muscle function and mass (8). Epidemiological data indicate that approximately 10%–16% of the global elderly population is affected by sarcopenia, with a significantly higher prevalence among patients with malignancies or critical illnesses (9). Numerous studies have identified sarcopenia as an important factor in poor postoperative prognosis in various cancers (10–12). In addition, recent studies have found that the state of inflammation is also related to tumor prognosis (13–16). The inflammatory response is generally evaluated using several biomarkers, including the neutrophil-to-lymphocyte ratio (NLR), platelet-to-lymphocyte ratio (PLR), lymphocyte-to-monocyte ratio (LMR), and C-reactive protein-to-albumin ratio (CAR). Reports indicate that a state of inflammation often coexists with sarcopenia, and together they affect patients’ quality of life (17, 18). The mechanism underlying this coexistence may involve the systemic inflammatory response affecting patients’ metabolism, and thereby accelerating skeletal muscle breakdown. This breakdown, in turn, generates local inflammation, leading to a vicious cycle of deteriorating systemic muscle function and quality (17, 18).

Although previous studies have individually investigated the prognostic impact of sarcopenia or systemic inflammatory status in patients undergoing PD, several critical knowledge gaps remain. First, existing evidence regarding the association between sarcopenia, post-PD complications, and OS is inconsistent across cohorts (19–26), partly due to varying diagnostic criteria and limited sample sizes. Second, while systemic inflammation is known to exacerbate skeletal muscle depletion, most studies have evaluated sarcopenia and inflammatory markers as isolated predictors rather than integrated prognostic factors. Consequently, whether combining preoperative sarcopenia with systemic inflammatory markers (such as NLR or CAR) can yield a synergistic or superior prognostic stratification for long-term OS in post-PD patients remains largely unexplored. To address these gaps, this study systematically evaluates the predictive value of preoperative sarcopenia, individual inflammatory markers, and, more importantly, their novel combination in a well-defined cohort of patients undergoing PD.

## 2 Methods

### 2.1 Patients

This retrospective study included 318 patients who underwent pancreaticoduodenectomy at the Second Affiliated Hospital of Chongqing Medical University between February 2019 and March 2025. The inclusion criteria were as follows: (1) Patients underwent PD surgery at our hospital between February 2019 and March 2025; (2) availability of abdominal computed tomography (CT) images and routine preoperative hematological test results obtained at our hospital within one month prior to surgery; (3) a preoperative Child-Pugh score of A or B. The exclusion criteria were: (1) evidence of distant tumor metastasis confirmed by preoperative examination; (2) a preoperative Child-Pugh score of C; (3) missing key clinical data (e.g., height, CRP).

All procedures performed in this study involving human participants complied with the ethical standards of the hospital and the National Research Council and with the 1964 Helsinki Declaration and its later amendments or comparable ethical standards. This study was approved by the Ethics Review Committee of the Second Affiliated Hospital of Chongqing Medical University (Ethics No: (2025)1099). Due to the retrospective nature of the study, the requirement for written informed consent from patients was waived.

### 2.2 Sarcopenia marker

Although the diagnosis of sarcopenia involves multiple approaches, CT is considered the gold standard for non-invasive assessment of body composition and muscle mass (27). The cross-sectional area of skeletal muscle at the L3 level (third lumbar vertebra) serves as a reliable indicator for assessing total skeletal muscle mass. This study employed SliceOmatic 5.0 software to calculate the combined cross-sectional area of the psoas major, erector spinae, quadratus lumborum, transversus abdominis, rectus abdominis, and combined internal and external oblique muscles at the L3 level as the Skeletal Muscle Area (SMA). The CT threshold range for muscle tissue was set at −29 to 150 HU. The total SMA (cm^2^) of all muscle tissues on the L3 cross-section was measured three consecutive times, and the mean value was used for analysis. The SMI was calculated using the following formula: Skeletal Muscle Index (SMI) = SMA (cm^2^) at L3 cross-section/Height^2^ (m^2^). In this study, sarcopenia was defined based on low skeletal muscle mass according to Martin criteria (28), namely: for males, SMI < 53.00 cm^2^/m^2^ (if BMI ≥ 25 kg/m^2^) or SMI < 43.00 cm^2^/m^2^ (if BMI < 25 kg/m^2^); for females, SMI < 41.00 cm^2^/m^2^. The CT image segmentation and SMA calculations were independently conducted by two trained researchers (including a clinical radiologist with >5 years of experience in abdominal imaging). Both observers strictly applied a blinded approach to all experimental data, post-PD complications, and survival outcomes.

### 2.3 Data collection

The clinical data were retrospectively collected and categorized as follows: (1) Demographic characteristics including age, gender, BMI (Body Mass Index), SMA and SMI. (2) Preoperative and intraoperative laboratory data: monocytes, lymphocytes, neutrophils, platelets (PLT), albumin (ALB), C-reactive protein (CRP), CA19-9 (Carbohydrate Antigen 19-9), CA125 (Cancer Antigen 125), CEA (Carcinoembryonic Antigen), intraoperative blood loss, and lymph node metastasis status. (3) Inflammatory marker indices comprised PLR, LMR, NLR, and CAR. (4) Outcome measures comprised primary and secondary endpoints. The primary endpoint was mortality. Secondary endpoints included postoperative complications, readmission within 30 days, and reoperation within 30 days. The postoperative complications included in this study are: delayed gastric emptying (DGE), postoperative bile fistula (PBF), postoperative pancreatic fistula (POPF), and postoperative haemorrhage. The diagnostic criteria for DGE adopted in this study align with those proposed by Wente et al. (29). The diagnostic criteria for PBF employed in this study are consistent with those proposed by Koch et al. (30). The diagnostic criteria for POPF employed in this study are consistent with those established by Bassi et al. (31). The diagnostic criteria for postoperative haemorrhage employed in this study are consistent with those proposed by Wente et al. (32).

### 2.4 Follow-up

All patients enrolled in this study were followed up via outpatient visits or telephone calls. Patients underwent follow-up examinations 3–6 months after undergoing PD surgery. OS was defined as the time from surgery to death or the last follow-up visit. The follow-up period for this study concluded on August 10, 2025.

### 2.5 Statistical analyses

This study employed SPSS 27.0 software for statistical analysis. Continuous variables were presented as mean ± standard deviation (SD) or median (interquartile range, IQR), while categorical variables were expressed as frequency (%). For intergroup comparisons, continuous variables were analyzed using Student's *t*-test or the Mann–Whitney *U* test, and categorical variables were assessed using the chi-square test or Fisher's exact test. The diagnostic performance of clinical indicators was evaluated using the area under the receiver operating characteristic (ROC) curve, with optimal cutoff values determined by the maximum Youden index. OS was estimated using the Kaplan–Meier method, and differences between groups were compared via the log-rank test. To identify independent predictors, variables with *P* < 0.10 in the univariate analysis were entered into multivariable Logistic regression models (for complications) and Cox proportional hazards models (for OS). Before conducting multivariable analysis, VIF and tolerance values were used to assess potential multicollinearity among the candidate covariates. Variables with interdependencies were excluded to prevent multicollinearity bias. A VIF value less than 5 was considered an indication that there was no significant multicollinearity. Before constructing the multivariable Cox proportional hazards regression models, the proportional hazards assumption was strictly tested for all entering variables using the time-dependent covariate method. Statistical significance was defined as a two-tailed *P* < 0.05. Variables with *P* < 0.10 in univariate analysis were considered for multivariable adjustment, which was strictly constrained by the events-per-variable (EPV) rule. Multivariable modeling was restricted to outcomes with adequate events, whereas analyses for rare complications were restricted to univariate screening to avoid separation bias. Given the significant differences between benign and malignant diseases in terms of biological behavior and long-term natural course, this study conducted a subgroup analysis of survival data for malignant diseases to account for heterogeneity across disease types.

## 3 Result

### 3.1 Patient characteristics

According to the inclusion and exclusion criteria, a total of 318 eligible patients who underwent PD at the Second Affiliated Hospital of Chongqing Medical University were enrolled in this study. The cohort consisted of 196 males (61.6%) and 122 females (38.4%), with an average age of 62.10±11.43 years. Sarcopenia was identified in 196 patients (61.6%). The comprehensive clinical characteristics of the study population are summarized in Table 1.

**Table 1:** Demographic and clinical characteristics of patients.

Variables		Mean ± SD or rate	Range
Age (years)		62.10 ± 11.43	26–88
SEX (male/female)		196 (61.64%)/122 (38.36%)	
Etiology (benign/malignant)		27 (8.49%)/291 (91.51%)	
BMI (kg/m^2^)		22.82 ± 3.09	15.35–40.40
SMI (cm^2^/m^2^)		42.46 ± 7.81	22.17–76.33
Monocyte (10^9^/L)		0.53 ± 0.23	0.06–1.55
Lymphocyte (10^9^/L)		1.35 ± 0.55	0.29–3.43
Neutrophils (10^9^/L)		4.62 ± 2.47	1.08–18.29
PLT (10^9^/L)		239.49 ± 88.10	68–674
ALB (g/L)		38.16 ± 4.49	23.60–51.50
CRP (mg/L)		17.87 ± 28.20	2–200
PLR		203.98 ± 109.43	25.19–979.31
LMR		2.96 ± 2.12	0.54–30.83
NLR		4.17 ± 4.14	0.69–54.83
CAR		0.51 ± 0.89	0.04–7.48
CA19-9 (U/mL)		264.16 ± 359.39	0.3–1,000
CA125 (U/mL)		28.45 ± 50.43	3.11–705.07
CEA (ng/mL)		4.81 ± 20.31	0.20–309.30
Operative blood loss (mL)		377.74 ± 291.06	50–2,300
SMA (cm^2^)		112.28 ± 25.80	51.88–205.30
Lymph node metastasis		84 (26.4%)	
Postoperative chemotherapy (with or without)		103 (32.39%)/215 (67.61%)	
Sarcopenia		196 (61.64%)	
Complication	DGE	164 (51.57%)	
	PBF	6 (1.89%)	
	POPF	92 (28.93%)	
	Postoperative Hemorrhage	51 (16.04%)	
Readmission within 30 days		31 (9.75%)	
Repeat surgery within 30 days		9(2.83%)	

BMI, body mass index; SMI, skeletal muscle index; PLT, platelet; ALB, albumin; CRP, C-reactive protein; PLR, platelet to lymphocyte ratio; LMR, lymphocyte to monocyte ratio; NLR, neutrophil to lymphocyte ratio; CAR, C-reactive protein to albumin ratio; CA19-9, carbohydrate antigen19-9; CA125, cancer antigen 125; CEA, carcinoembryonic antigen; SMA, skeletal muscle area; DGE, delayed gastric emptying; PBF, postoperative bile fistula; POPF, postoperative pancreatic fistula.

### 3.2 Comparison between the sarcopenia and Non-sarcopenia groups

The clinical characteristics of patients stratified by sarcopenia status are summarized in Table 3. Compared with the non-sarcopenia group, patients with sarcopenia were significantly older (64.84±10.29vs.57.70±11.83years,P<0.001) and had a lower proportion of males (53.6% vs. 74.6%, *P* < 0.001). As expected, both BMI and SMI were significantly lower in the sarcopenia group (*P* < 0.001). Regarding laboratory findings, the sarcopenia group exhibited significantly lower lymphocyte counts (P=0.009) but higher CRP levels (P=0.008) and a greater prevalence of high CAR 34.2%vs.19.7%,P=0.008. Although CA19-9 and CA125 levels showed statistical differences between the two groups (*P* < 0.05), no significant differences were observed in etiology, monocytes, neutrophils, PLT, ALB, PLR, LMR, NLR, CEA, intraoperative blood loss, lymph node metastasis or postoperative chemotherapy (*P* > 0.05; Table 3).

**Table 2:** Area under the ROC curve for systemic inflammatory markers in relation to mortality.

Variables	Area under the curve	Cutoff value	Sensitivity	Specificity	*P* value
NLR	0.608	2.705	0.768	0.436	**0** **.** **004**
CAR	0.588	0.372	0.415	0.758	**0**.**018**
PLR	0.545	139.108	0.793	0.322	0.223
LMR	0.445	0.807	0.988	0.025	0.140

NLR, neutrophil to lymphocyte ratio; CAR, C-reactive protein to albumin ratio; PLR, platelet to lymphocyte ratio; LMR, lymphocyte to monocyte ratio.

The bold values indicate that *P* < 0.10.

**Table 3:** Preoperative general characteristics of the sarcopenia group and Non-sarcopenia group.

Variables	Sarcopenia Group(*n* = 196)	Non-sarcopenia Group(*n* = 122)	*P* value
Age (years)	64.84 ± 10.29	57.70 ± 11.83	**<0.001**
SEX (male/female)	105 (53.6%)	91 (74.6%)	**<0.001**
Etiology (benign/malignant)	18 (9.18%)/178 (90.82%)	9 (7.38%)/113 (92.62%)	0.681
BMI (kg/m^2^)	22.32 ± 3.31	23.62 ± 2.51	**<0.001**
SMI (cm^2^/m^2^)	38.21 ± 5.65	49.23 ± 5.71	**<0.001**
Monocyte (10^9^/L)	0.53 ± 0.24	0.53 ± 0.22	0.714
Lymphocyte (10^9^/L)	1.29 ± 0.52	1.45 ± 0.57	**0**.**009**
Neutrophils (10^9^/L)	4.73 ± 2.61	4.43 ± 2.23	0.257
PLT (10^9^/L)	239.69 ± 92.57	239.16 ± 80.79	0.58
ALB (g/L)	37.83 ± 4.78	38.68 ± 3.95	0.1
CRP (mg/L)	20.39 ± 30.95	13.81 ± 22.65	**0**.**008**
PLR >139.108	145 (74.0%)	80 (65.6%)	0.14
LMR >0.807	191 (97.4%)	120 (98.4%)	0.884
NLR >2.705	127 (64.8%)	69 (56.6%)	0.177
CAR >0.372	67 (34.2%)	24 (19.7%)	**0**.**008**
CA19-9 (U/mL)	288.55 ± 364.74	224.98 ± 348.54	**0**.**036**
CA125 (U/mL)	29.01 ± 37.30	27.54 ± 66.47	**0**.**034**
CEA (ng/mL)	5.09 ± 22.45	4.37 ± 16.38	0.793
Operative blood loss (mL)	393.11 ± 282.05	353.03 ± 304.52	0.113
SMA (cm^2^)	100.37 ± 20.55	131.25 ± 21.67	**<0.001**
Lymph node metastasis	49 (25.0%)	35 (28.7%)	0.552
Postoperative chemotherapy (with or without)	57 (29.08%)/139 (70.92%)	46 (37.70%)/76 (62.30%)	0.139

BMI, body mass index; SMI, skeletal muscle index; PLT, platelet; ALB, albumin; CRP, C-reactive protein; PLR, platelet to lymphocyte ratio; LMR, lymphocyte to monocyte ratio; NLR, neutrophil to lymphocyte ratio; CAR, C-reactive protein to albumin ratio; CA19-9, carbohydrate antigen19-9; CA125, cancer antigen 125; CEA, carcinoembryonic antigen; SMA, skeletal muscle area.

The bold values indicate that *P* < 0.10.

### 3.3 Survival analysis

**Figure 1:**
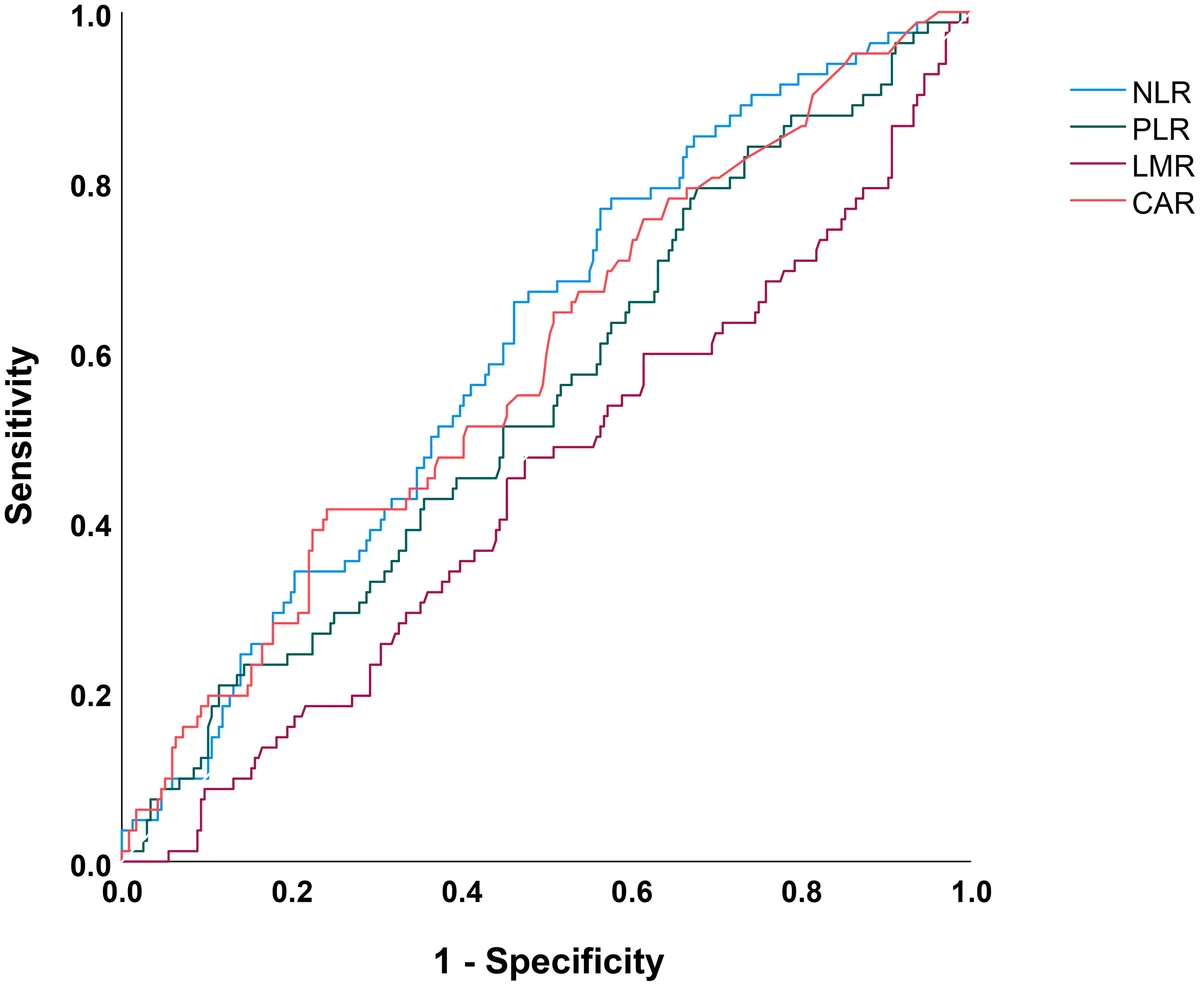
ROC curve analysis of systemic inflammatory markers for predicting mortality. NLR, neutrophil to lymphocyte ratio, PLR, platelet to lymphocyte ratio, LMR, lymphocyte to monocyte ratio, CAR, C-reactive protein to albumin ratio.

ROC curves were constructed to evaluate the predictive performance of systemic inflammatory markers for mortality (Figure 1, Table 2). The AUC values for NLR and CAR were 0.608 (95% CI: 0.540–0.675, *P* < 0.05) and 0.588 (95% CI: 0.517–0.659, *P* < 0.05), respectively, indicating a statistically significant but modest individual predictive value. In contrast, the AUC for PLR and LMR did not reach statistical significance, with values of 0.545 (95% CI: 0.474–0.617, *P* = 0.223) and 0.445 (95% CI: 0.372–0.518, *P* = 0.140), respectively. Optimal cutoff values for CAR, NLR, PLR, and LMR were determined to be 0.372, 2.705, 139.108, and 0.807, respectively, based on the maximum Youden index derived from the ROC curves.

**Figure 2:**
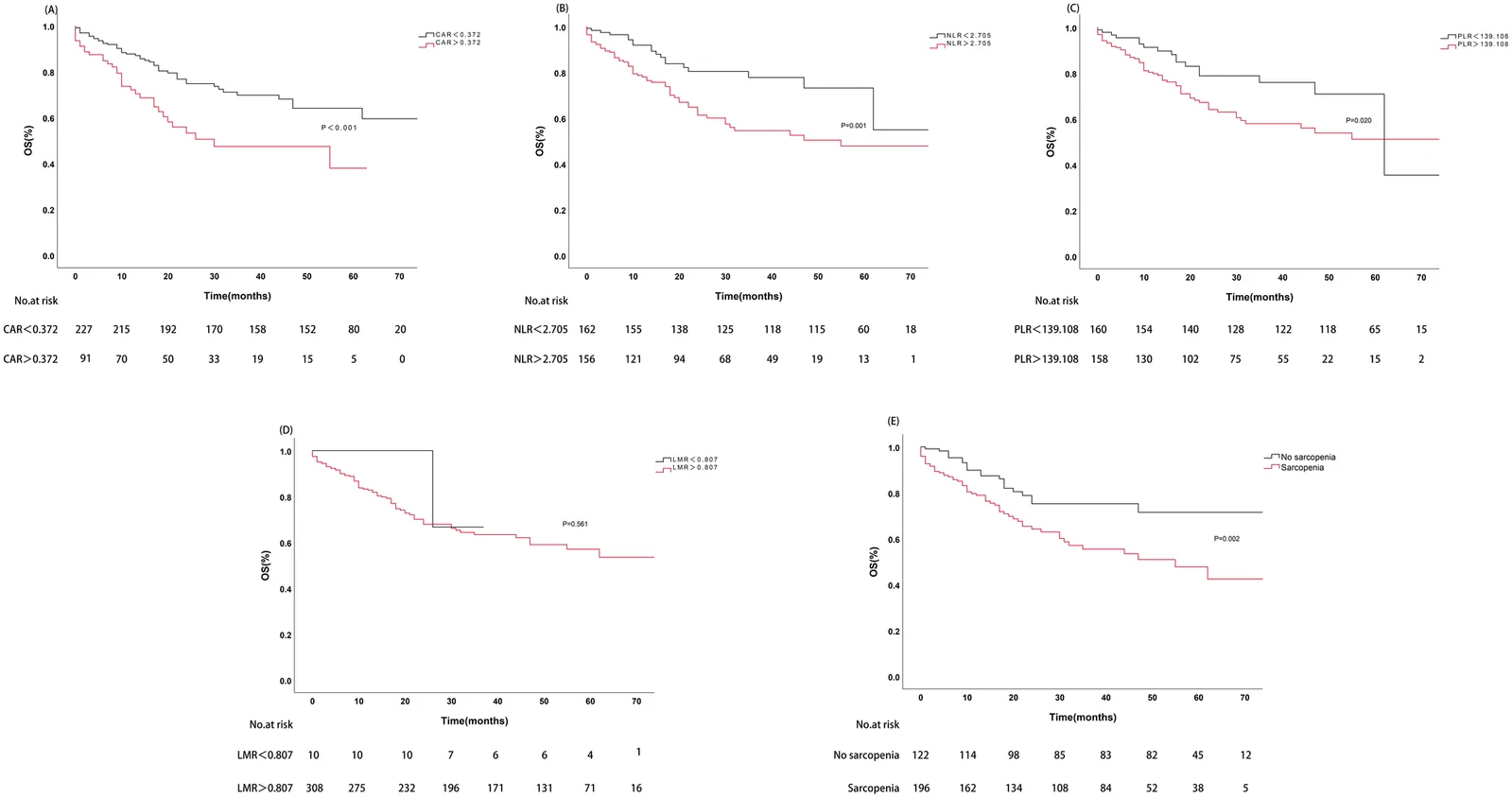
Kaplan–Meier curves according to CAR, NLR, PLR, LMR and sarcopenia. **(A)** Kaplan–Meier survival curves for CAR; **(B)** Kaplan–Meier survival curves for NLR; **(C)** Kaplan–Meier survival curves for PLR; **(D)** Kaplan–Meier survival curves for LMR; **(E)** Kaplan–Meier survival curves for sarcopenia.

In this study, 82 patients died, 200 survived, and 36 were lost to follow-up. Kaplan–Meier survival analysis (Figure 2) demonstrated that patients with sarcopenia had a significantly shorter median OS compared to non-sarcopenic patients (47 vs. 61 months, *P* = 0.002). Similarly, significantly reduced median OS was observed in the high-CAR group compared to the low-CAR group (36 vs. 57 months, *P* < 0.001), the high-NLR group compared to the low-NLR group (47 vs. 60 months, *P* = 0.001), and the high-PLR group compared to the low-PLR group (49 vs. 56 months, *P* = 0.020). In contrast, no statistically significant difference in OS was found between the high-LMR and low-LMR groups (52 vs. 33 months, *P* = 0.561).

Using postoperative OS as the primary endpoint, univariate Cox regression analysis identified that advanced age, lymph node metastasis, preoperative sarcopenia, and elevated levels of neutrophils, CRP, PLR, NLR, CAR, CA19-9, and CA125 along with decreased levels of lymphocytes and ALB were significantly associated with poor OS (*P* < 0.10). Subsequently, multivariable Cox regression analysis demonstrated that age (HR = 1.023; 95% CI: 1.000–1.046; *P* = 0.049); NLR (HR = 1.818; 95% CI: 1.070–3.090; *P* = 0.027); CAR (HR = 1.666; 95% CI: 1.061–2.617; *P* = 0.027); CA199 (HR = 1.000; 95% CI: 1.000–1.001; *P* = 0.041); lymph node metastasis (HR = 2.013; 95% CI: 1.254–3.299; *P* = 0.004) and sarcopenia (HR = 1.935; 95% CI: 1.158–3.234; *P* = 0.012) remained independent prognostic factors for OS in patients following PD (Table 4). Our subgroup analysis of patients with malignant tumors found that sarcopenia was not an independent risk factor for OS (*P* = 0.203, Table 5).

**Table 4:** Univariate and multivariate analysis of OS.

Variables	Univariate analysis		Multivariate analysis	
	HR(95% CI)	*P* value	HR (95% CI)	*P* value
Age (years)	1.037 (1.016–1.058)	**<0.001**	1.023 (1.000–1.046)	**0**.**049**
SEX (male/female)	1.191 (0.760–1.869)	0.446		
Etiology (benign/malignant)	2.981 (0.939–9.467)	0.064		
BMI (kg/m^2^)	1.030 (0.959–1.105)	0.416		
SMI (cm^2^/m^2^)	0.998 (0.970–1.026)	0.868		
Monocyte (10^9^/L)	1.519 (0.624–3.700)	0.357		
Lymphocyte (10^9^/L)	0.667 (0.431–1.033)	**0**.**070**		
Neutrophils (10^9^/L)	1.103 (1.027–1.184)	**0**.**007**		
PLT (10^9^/L)	1.001 (0.998–1.003)	0.537		
ALB (g/L)	0.944 (0.902–0.989)	**0**.**015**		
CRP (mg/L)	1.007 (1.001–1.013)	**0**.**020**		
PLR	1.862 (1.019–3.180)	**0**.**023**		
LMR	1.773 (0.246–12.754)	0.569		
NLR	2.263 (1.345–3.782)	**0**.**002**	1.818 (1.070–3.090)	**0**.**027**
CAR	2.180 (1.402–3.390)	**<0.001**	1.666 (1.061–2.617)	**0**.**027**
CA19-9 (U/mL)	1.001 (1.000–1.001)	**0**.**001**	1.000 (1.000–1.001)	**0**.**041**
CA125 (U/mL)	1.003 (1.000–1.006)	**0**.**027**	1.002 (0.998–1.005)	0.273
CEA (ng/mL)	0.998 (0.986–1.010)	0.722		
Operative blood loss (mL)	1.000 (0.999–1.001)	0.901		
SMA (cm^2^)	0.999 (0.991–1.008)	0.869		
Lymph node metastasis	2.141 (1.352–3.390)	**0**.**001**	2.013 (1.254–3.299)	**0**.**004**
Postoperative chemotherapy (with or without)	0.730 (0.451–1.183)	0.201		
Sarcopenia	2.141 (1.303–3.518)	**0**.**003**	1.935(1.158–3.234)	**0**.**012**

BMI, body mass index; SMI, skeletal muscle index; PLT, platelet; ALB, albumin; CRP, C-reactive protein; PLR, platelet to lymphocyte ratio; LMR, lymphocyte to monocyte ratio; NLR, neutrophil to lymphocyte ratio; CAR, C-reactive protein to albumin ratio; CA19-9, carbohydrate antigen19-9; CA125, cancer antigen 125; CEA, carcinoembryonic antigen; SMA, skeletal muscle area.

The bold values indicate that *P* < 0.10.

**Table 5:** Univariate and multivariate analysis of COX in patients with malignant tumors.

Variables	Univariate analysis		Multivariate analysis	
	HR(95% CI)	*P* value	HR (95% CI)	*P* value
Age (years)	1.031 (1.009–1.054)	**0** **.** **006**	1.032 (1.008–1.058)	**0**.**010**
SEX (male/female)	1.503 (0.935–2.418)	**0**.**092**		
Etiology: Cholangiocarcinoma		**0**.**067**		**<0.001**
Pancreatic Neoplasms	1.475 (0.747–2.911)	0.263	2.533 (1.197–5.360)	**0**.**015**
Periampullary carcinoma	0.969 (0.416–2.038)	0.934	1.114 (0.499–2.486)	0.792
Duodenal Neoplasms	0.624 (0.275–1.414)	0.258	0.602 (0.246–1.473)	0.266
TNM I		0.117		**0**.**004**
II	1.656 (0.979–2.801)	0.060	1.997 (1.124–3.548)	**0**.**018**
III	1.676 (0.920–3.054)	0.091	3.053 (1.551–6.011)	**0**.**001**
BMI (kg/m^2^)	1.030 (0.955–1.100)	0.445		
SMI (cm^2^/m^2^)	1.005 (0.977–1.034)	0.743		
Monocyte (10^9^/L)	1.779 (0.675–4.691)	0.244		
Lymphocyte (10^9^/L)	0.671 (0.417–1.080)	0.100		
Neutrophils (10^9^/L)	1.113 (1.036–1.195)	**0**.**003**		
PLT (10^9^/L)	1.000 (0.998–1.003)	0.830		
ALB (g/L)	0.963 (0.916–1.014)	0.150		
CRP (mg/L)	1.009 (1.003–1.016)	**0**.**002**		
PLR	1.951 (1.136–3.349)	**0**.**015**		
LMR	1.515 (0.211–10.910)	0.680		
NLR	2.625 (1.469–4.689)	**0**.**001**		
CAR	2.107 (1.335–3.325)	**0**.**001**		
CA19-9 (U/mL)	1.001 (1.000–1.001)	**0**.**012**		
CA125 (U/mL)	1.003 (1.000–1.006)	**0**.**065**		
CEA (ng/mL)	0.997 (0.985–1.010)	0.657		
Operative blood loss (mL)	1.000 (0.999–1.001)	0.778		
SMA(cm^2^)	1.003 (0.994–1.011)	0.548		
Lymph node metastasis	1.814 (1.136–2.897)	**0**.**013**		
Postoperative chemotherapy				
(with or without)	0.608 (0.372–0.992)	**0**.**046**	0.705 (0.422–1.178)	0.182
Sarcopenia	1.448 (0.904–2.319)	0.123	1.378(0.841–2.258)	0.203

BMI, body mass index; SMI, skeletal muscle index; PLT, platelet; ALB, albumin; CRP, C-reactive protein; PLR, platelet to lymphocyte ratio; LMR, lymphocyte to monocyte ratio; NLR, neutrophil to lymphocyte ratio; CAR, C-reactive protein to albumin ratio; CA19-9, carbohydrate antigen19-9; CA125, cancer antigen 125; CEA, carcinoembryonic antigen; SMA, skeletal muscle area.

The bold values indicate that *P* < 0.10.

### 3.4 Influencing factors of complication

This study evaluated the potential associations between sarcopenia, systemic inflammatory markers, and secondary outcome measures. No significant correlations were observed between sarcopenia or inflammatory markers and the occurrence of postoperative complications, including DGE, PBF, POPF, and postoperative hemorrhage (Tables 6–9; *P* > 0.05). Similarly, neither sarcopenia nor inflammatory markers were significantly associated with 30-day readmission or 30-day reoperation rates (Tables 10, 11; *P* > 0.05).

**Table 6:** Univariate and multivariate analysis of DGE.

Variables	Univariate analysis		Multivariate analysis	
	OR(95% CI)	*P* value	OR (95% CI)	*P* value
Age (years)	0.997 (0.978–1.017)	0.787		
SEX (male/female)	0.804 (0.511–1.226)	0.347		
BMI (kg/m^2^)	0.976 (0.909–1.049)	0.510		
SMI (cm^2^/m^2^)	0.953 (0.925–0.982)	**0** **.** **002**	0.933 (0.858–1.015)	0.106
Monocyte (10^9^/L)	0.415 (0.157–1.099)	**0**.**077**	0.364 (0.126–1.046)	0.061
Lymphocyte (10^9^/L)	0.916 (0.612–1.373)	0.672		
Neutrophils (10^9^/L)	1.054 (0.962–1.156)	0.257		
PLT (10^9^/L)	1.004 (1.001–1.007)	**0**.**002**	1.171 (0.670–2.048)	0.579
ALB (g/L)	1.004 (0.956–1.055)	0.862		
CRP (mg/L)	1.000 (0.992–1.008)	0.995		
PLR	1.842 (1.128–3.007)	**0**.**015**	1.171 (0.670–2.048)	0.579
LMR	2.718 (0.519–14.222)	0.236		
NLR	1.168 (0.743–1.836)	0.501		
CAR	1.286 (0.789–2.097)	0.313		
CA19-9 (U/mL)	1.000 (1.000–1.001)	0.238		
CA125 (U/mL)	1.001 (0.997–1.006)	0.636		
CEA (ng/mL)	0.990 (0.971–1.010)	0.323		
Operative blood loss (mL)	1.001 (1.000–1.001)	0.110		
SMA (cm^2^)	0.988 (0.980–0.997)	**0**.**009**	1.011 (0.988–1.033)	0.349
Lymph node metastasis	1.115 (0.677–1.838)	0.669		
Sarcopenia	1.700 (1.078–2.682)	**0**.**023**	1.116(0.576–2.163)	0.745

The bold values indicate that *P* < 0.10.

**Table 7:** Univariate and multivariate analysis of PBF.

Variables	Univariate analysis		Multivariate analysis	
	OR(95% CI)	*P* value	OR (95% CI)	*P* value
Age (years)	0.990 (0.918–1.068)	0.797		
SEX (male/female)	0.993 (0.154–5.663)	0.940		
BMI (kg/m^2^)	1.087 (0.844–1.402)	0.518		
SMI (cm^2^/m^2^)	1.012 (0.906–1.131)	0.834		
Monocyte (10^9^/L)	0.003 (0.000–2.040)	**0** **.** **081**		
Lymphocyte (10^9^/L)	0.891 (0.168–4.730)	0.893		
Neutrophils (10^9^/L)	0.455 (0.193–1.071)	**0**.**071**		
PLT (10^9^/L)	0.986 (0.971–1.002)	**0**.**082**		
ALB (g/L)	1.053 (0.865–1.281)	0.607		
CRP (mg/L)	0.826 (0.543–1.255)	0.370		
PLR	0.269 (0.044–1.637)	0.154		
LMR	0.520 (0.31–3.242)	0.999		
NLR	0.409 (0.067–2.483)	0.331		
CAR	0.243 (0.134–2.472)	0.874		
CA19-9 (U/mL)	0.997 (0.991–1.003)	0.324		
CA125 (U/mL)	0.980 (0.917–1.047)	0.549		
CEA (ng/mL)	0.929 (0.616–1.401)	0.725		
Operative blood loss (mL)	1.001 (0.999–1.003)	0.223		
SMA (cm^2^)	1.006 (0.973–1.040)	0.708		
Lymph node metastasis	0.693 (0.076–6.288)	0.744		
Sarcopenia	2.521 (0.278–22.821)	0.411		

The bold values indicate that *P* < 0.10.

**Table 8:** Univariate and multivariate analysis of POPF.

Variables	Univariate analysis		Multivariate analysis	
	OR(95% CI)	*P* value	OR (95% CI)	*P* value
Age (years)	1.002 (0.982–1.023)	0.847		
SEX (male/female)	1.205 (0.746–1.945)	0.446		
BMI (kg/m^2^)	1.053 (0.974–1.139)	0.195		
SMI (cm^2^/m^2^)	1.011 (0.980–1.042)	0.499		
Monocyte (10^9^/L)	2.269 (0.773–6.660)	0.136		
Lymphocyte (10^9^/L)	1.631 (1.029–2.585)	**0** **.** **037**	1.194 (0.680–2.096)	0.573
Neutrophils (10^9^/L)	0.941 (0.859–1.032)	0.197		
PLT (10^9^/L)	1.002 (0.999–1.005)	0.135		
ALB (g/L)	0.980 (0.930–1.033)	0.455		
CRP (mg/L)	0.991 (0.983–0.999)	**0**.**034**	0.994 (0.985–1.002)	0.152
PLR	0.679 (0.398–1.159)	0.156		
LMR	1.559 (0.343–7.099)	0.566		
NLR	0.583 (0.325–0.889)	**0**.**016**	0.746 (0.396–1.402)	0.362
CAR	0.699 (0.420–1.162)	0.167		
CA19-9 (U/mL)	0.999 (0.998–1.000)	**0**.**006**	0.999 (0.999–1.000)	**0**.**027**
CA125 (U/mL)	1.000 (0.995–1.005)	0.981		
CEA (ng/mL)	0.994 (0.983–1.006)	0.345		
Operative blood loss (mL)	1.000 (1.000–1.001)	0.350		
SMA (cm^2^)	1.004 (0.995–1.013)	0.423		
Lymph node metastasis	0.895 (0.528–1.515)	0.679		
Sarcopenia	1.006 (0.622–1.629)	0.980		

The bold values indicate that *P* < 0.10.

**Table 9:** Univariate and multivariate analysis of postoperative hemorrhage.

Variables	Univariate analysis		Multivariate analysis	
	OR(95% CI)	*P* value	OR (95% CI)	*P* value
Age (years)	0.992 (0.969–1.015)	0.484		
SEX (male/female)	1.603 (0.906–2.836)	0.105		
BMI (kg/m^2^)	1.021 (0.938–1.112)	0.635		
SMI (cm^2^/m^2^)	1.002 (0.968–1.037)	0.913		
Monocyte (10^9^/L)	0.654 (0.198–2.161)	0.487		
Lymphocyte (10^9^/L)	1.270 (0.791–2.042)	0.323		
Neutrophils (10^9^/L)	1.025 (0.924–1.136)	0.642		
PLT (10^9^/L)	1.002 (0.999–1.005)	0.188		
ALB (g/L)	1.038 (0.978–1.101)	0.224		
CRP (mg/L)	0.999 (0.989–1.009)	0.824		
PLR	0.875 (0.493–1.555)	0.649		
LMR	0.700 (0.133–3.686)	0.673		
NLR	0.961 (0.532–1.157)	0.750		
CAR	1.090 (0.609–1.949)	0.772		
CA19-9 (U/mL)	1.000 (1.000–1.001)	0.279		
CA125 (U/mL)	1.003 (0.998–1.008)	0.195		
CEA (ng/mL)	0.996 (0.978–1.015)	0.686		
Operative blood loss (mL)	1.002 (1.001–1.003)	**<0.001**	1.002 (1.000–1.003)	**<0.001**
SMA (cm^2^)	1.006 (0.996–1.016)	0.256		
Lymph node metastasis	0.867 (0.469–1.601)	0.647		
Sarcopenia	1.358 (0.776–2.376)	0.284		

The bold values indicate that *P* < 0.10.

**Table 10:** Univariate and multivariate analyses of readmission within 30 days.

Variables	Univariate analysis		Multivariate analysis	
	OR(95% CI)	*P* value	OR (95% CI)	*P* value
Age (years)	0.999 (0.967–1.031)	0.932		
SEX (male/female)	2.292 (0.956–5.496)	**0** **.** **063**	1.790 (0.502–6.388)	0.370
BMI (kg/m^2^)	1.089 (0.973–1.219)	0.136		
SMI (cm^2^/m^2^)	1.078 (1.029–1.129)	**0**.**001**	1.165 (1.006–1.349)	**0**.**041**
Monocyte (10^9^/L)	2.904 (0.689–12.246)	0.147		
Lymphocyte (10^9^/L)	1.552 (0.808–2.876)	0.194		
Neutrophils (10^9^/L)	1.151 (1.206–1.291)	**0**.**016**	1.191 (1.053–1.347)	**0**.**005**
PLT (10^9^/L)	1.002 (0.998–1.006)	0.289		
ALB (g/L)	1.031 (0.949–1.120)	0.465		
CRP (mg/L)	1.005 (0.995–1.016)	0.388		
PLR	0.623 (0.289–1.341)	0.226		
LMR	0.225 (0.156–2.001)	0.999		
NLR	1.147 (0.529–2.484)	0.729		
CAR	1.423 (0.653–3.103)	0.375		
CA19-9 (U/mL)	1.000 (0.999–1.001)	0.963		
CA125 (U/mL)	0.990 (0.972–1.009)	0.320		
CEA (ng/mL)	0.942 (0.810–1.096)	0.440		
Operative blood loss (mL)	1.001 (1.000–1.002)	**0**.**057**	1.001 (1.000–1.002)	**0**.**022**
SMA (cm^2^)	1.020 (1.006–1.035)	**0**.**005**	0.969 (0.925–1.016)	0.193
Lymph node metastasis	1.156 (0.510–2.623)	0.728		
Sarcopenia	0.354 (0.165–0.757)	**0**.**007**	0.643(0.226–1.836)	0.410

The bold values indicate that *P* < 0.10.

**Table 11:** Univariate and multivariate analysis of reoperation within 30 days.

Variables	Univariate analysis		Multivariate analysis	
	OR(95% CI)	*P* value	OR (95% CI)	*P* value
Age (years)	0.975 (0.923–1.030)	0.362		
SEX (male/female)	1.253 (0.307–5.104)	0.753		
BMI (kg/m^2^)	1.079 (0.888–1.311)	0.447		
SMI (cm^2^/m^2^)	1.063 (0.985–1.147)	0.116		
Monocyte (10^9^/L)	0.505 (0.222–5.806)	0.671		
Lymphocyte (10^9^/L)	1.328 (0.424–4.158)	0.626		
Neutrophils (10^9^/L)	1.033 (0.810–1.318)	0.794		
PLT (10^9^/L)	1.003 (0.997–1.010)	0.291		
ALB (g/L)	0.983 (0.848–1.140)	0.821		
CRP (mg/L)	0.911 (0.778–1.065)	0.242		
PLR	3.392 (0.418–27.507)	0.253		
LMR	1.382 (0.837–2.476)	0.999		
NLR	2.222 (0.454–10.876)	0.324		
CAR	—	1.000		
CA19-9 (U/mL)	1.001 (0.999–1.003)	0.242		
CA125 (U/mL)	1.004 (0.997–1.010)	0.245		
CEA (ng/mL)	1.003 (0.981–1.026)	0.793		
Operative blood loss (mL)	1.002 (1.000–1.003)	**0** **.** **008**		
SMA (cm^2^)	1.019 (0.994–1.043)	0.132		
Lymph node metastasis	0.791 (0.161–3.885)	0.773		
Sarcopenia	0.536 (0.141–2.036)	0.360		

The bold values indicate that *P* < 0.10.

Furthermore, combined analyses of sarcopenia and inflammatory markers revealed no significant associations with any of the secondary outcomes evaluated (Tables 12–15; *P* > 0.05).

**Table 12:** DGE.

Variables (Sarcopenia and NLR/CAR/PLR/LMR status)	Multivariate analysis	
	OR (95% CI)	*P* value
Non-sarcopenia and a low NLR	Ref.	
Sarcopenia and a low NLR	1.244 (0.525–2.952)	0.620
Non-sarcopenia and a high NLR	1.213 (0.552–2.664)	0.631
Sarcopenia and a high NLR	1.264 (0.523–3.054)	0.602
Non-sarcopenia and a low CAR	Ref.	
Sarcopenia and a low CAR	1.063 (0.521–2.169)	0.866
Non-sarcopenia and a high CAR	1.182 (0.465–3.003)	0.726
Sarcopenia and a high CAR	1.437 (0.624–3.308)	0.394
Non-sarcopenia and a low PLR	Ref.	
Sarcopenia and a low PLR	1.274 (0.488–3.326)	0.620
Non-sarcopenia and a high PLR	1.342 (0.589–3.060)	0.484
Sarcopenia and a high PLR	1.413 (0.571–3.494)	0.455
Non-sarcopenia and a low LMR	Ref.	
Sarcopenia and a low LMR	0	0.999
Non-sarcopenia and a high LMR	0	0.999
Sarcopenia and a high LMR	0	0.999

**Table 13:** POPF.

Variables (Sarcopenia and NLR/CAR/PLR/LMR status)	Multivariate analysis	
	OR (95% CI)	*P* value
Non-sarcopenia and a low NLR	Ref.	
Sarcopenia and a low NLR	1.582 (0.684–3.659)	0.283
Non-sarcopenia and a high NLR	0.987 (0.411–2.370)	0.977
Sarcopenia and a high NLR	0.976 (0.423–2.254)	0.954
Non-sarcopenia and a low CAR	Ref.	
Sarcopenia and a low CAR	1.199 (0.670–2.147)	0.541
Non-sarcopenia and a high CAR	1.309 (0.449–3.811)	0.622
Sarcopenia and a high CAR	1.345 (0.585–3.094)	0.485
Non-sarcopenia and a low PLR	Ref.	
Sarcopenia and a low PLR	2.556 (0.977–6.690)	0.056
Non-sarcopenia and a high PLR	1.649 (0.681–3.996)	0.268
Sarcopenia and a high PLR	1.427 (0.614–3.317)	0.408
Non-sarcopenia and a low LMR	Ref.	
Sarcopenia and a low LMR	0.755 (0.022–25.674)	0.876
Non-sarcopenia and a high LMR	0.629 (0.029–13.739)	0.769
Sarcopenia and a high LMR	0.742 (0.035–15.646)	0.848

**Table 14:** Postoperative hemorrhage.

Variables (Sarcopenia and NLR/CAR/PLR/LMR status)	Multivariate analysis	
	OR (95% CI)	*P* value
Non-sarcopenia and a low NLR	Ref.	
Sarcopenia and a low NLR	1.116 (0.456–2.728)	0.810
Non-sarcopenia and a high NLR	0.819 (0.317–2.119)	0.681
Sarcopenia and a high NLR	1.165 (0.523–2.596)	0.709
Non-sarcopenia and a low CAR	Ref.	
Sarcopenia and a low CAR	1.078 (0.554–2.097)	0.825
Non-sarcopenia and a high CAR	0.542 (0.143–2.048)	0.366
Sarcopenia and a high CAR	1.281 (0.599–2.739)	0.523
Non-sarcopenia and a low PLR	Ref.	
Sarcopenia and a low PLR	2.249 (0.880–5.747)	0.090
Non-sarcopenia and a high PLR	1.249 (0.566–2.755)	0.582
Sarcopenia and a high PLR	0.939 (0.459–1.924)	0.864
Non-sarcopenia and a low LMR	Ref.	
Sarcopenia and a low LMR	0.336 (0.008–13.781)	0.565
Non-sarcopenia and a high LMR	0.326 (0.017–6.457)	0.462
Sarcopenia and a high LMR	0.431 (0.022–8.349)	0.578

**Table 15:** Readmitted within 30 days.

Variables (Sarcopenia and NLR/CAR/PLR/LMR status)	Multivariate analysis	
	OR (95% CI)	*P* value
Non-sarcopenia and a low NLR	Ref.	
Sarcopenia and a low NLR	0.594 (0.124–2.846)	0.515
Non-sarcopenia and a high NLR	0.878 (0.290–2.653)	0.817
Sarcopenia and a high NLR	0.591 (0.151–2.313)	0.450
Non-sarcopenia and a low CAR	Ref.	
Sarcopenia and a low CAR	0.594 (0.177–1.988)	0.398
Non-sarcopenia and a high CAR	1.084 (0.315–3.731)	0.899
Sarcopenia and a high CAR	0.779 (0.198–3.061)	0.720
Non-sarcopenia and a low PLR	Ref.	
Sarcopenia and a low PLR	1.015 (0.241–4.285)	0.984
Non-sarcopenia and a high PLR	1.008 (0.342–2.975)	0.988
Sarcopenia and a high PLR	0.479 (0.116–1.974)	0.308
Non-sarcopenia and a low LMR	Ref.	
Sarcopenia and a low LMR	0	1
Non-sarcopenia and a high LMR	0	0.999
Sarcopenia and a high LMR	0	0.999

DGE, delayed gastric emptying; PBF, postoperative bile fistula; POPF, postoperative pancreatic fistula; NLR, neutrophil to lymphocyte ratio; CAR, C-reactive protein to albumin ratio; PLR, platelet to lymphocyte ratio; LMR, lymphocyte to monocyte ratio.

### 3.5 Effect of sarcopenia combined with NLR or CAR on survival

To further evaluate the prognostic value of sarcopenia and inflammatory markers, multivariable Cox regression analysis was performed by incorporating combined variables. The results identified the “sarcopenia and high NLR” combination (HR = 5.155; 95% CI: 1.551–17.136; *P* = 0.007) and the “sarcopenia and high CAR” combination (HR = 2.808; 95% CI: 1.486–5.308; *P* = 0.001) as independent predictors of OS after PD (Table 16). When we performed a subgroup analysis of cancer patients, we found that “sarcopenia and high CAR” remained risk factors for OS after PD (HR = 2.193, 95% CI: 1.050–4.582, *P* = 0.037, Table 17).

**Table 16:** Multivariate analyses of overall survival including sarcopenia and NLR/CAR status.

Variables (Sarcopenia and NLR/CAR status)	Multivariate analysis	
	HR (95% CI)	*P* value
Non-sarcopenia and a low NLR	Ref.	
Sarcopenia and a low NLR	3.008 (0.867–10.472)	0.083
Non-sarcopenia and a high NLR	3,223 (0.926–11.215)	0.066
Sarcopenia and a high NLR	5.155 (1.551–17.136)	**0** **.** **007**
Non-sarcopenia and a low CAR	Ref.	
Sarcopenia and a low CAR	1.162 (0.424–3.183)	0.770
Non-sarcopenia and a high CAR	1.524 (0.824–2.817)	0.179
Sarcopenia and a high CAR	2.808 (1.486–5.308)	**0**.**001**

The bold values indicate that *P* < 0.10.

**Table 17:** Multivariate analysis of overall survival in patients with malignant tumors based on sarcopenia and NLR/CAR.

Variables (Sarcopenia and NLR/CAR status)	Multivariate analysis	
	HR (95% CI)	*P* value
Non-sarcopenia and a low NLR	Ref.	
Sarcopenia and a low NLR	2.508 (0.765–8.225)	0.129
Non-sarcopenia and a high NLR	2.276 (0.741–6.988)	0.151
Sarcopenia and a high NLR	2.844 (0.901–8.974)	0.075
Non-sarcopenia and a low CAR	Ref.	
Sarcopenia and a low CAR	1.500 (0.532–4.226)	0.443
Non-sarcopenia and a high CAR	1.445 (0.761–2.745)	0.260
Sarcopenia and a high CAR	2.193 (1.050–4.582)	**0** **.** **037**

NLR, neutrophil to lymphocyte ratio; CAR, C-reactive protein to albumin ratio.

The bold values indicate that *P* < 0.10.

**Figure 3:**
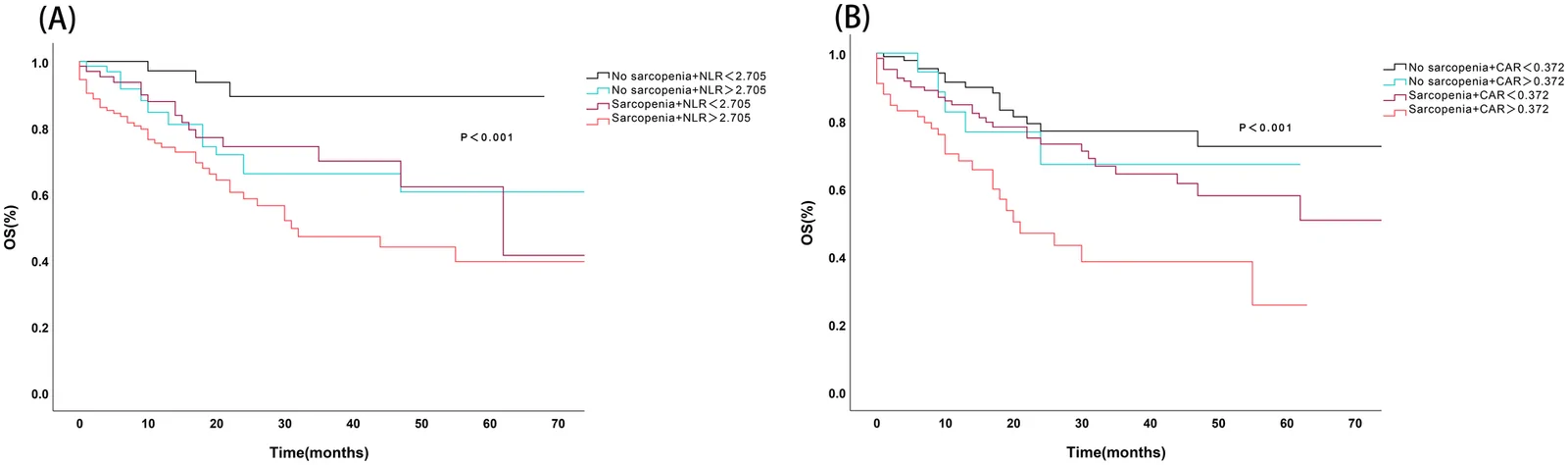
Kaplan–Meier curves according to sarcopenia, NLR and CAR. **(A)** Sarcopenia and a high NLR were identified as significant additive risk factors for poor survival (*P* < 0.001). **(B)** Sarcopenia and a high CAR were identified as significant additive risk factors for poor survival (*P* < 0.001).

Furthermore, patients were regrouped according to their sarcopenia and inflammatory marker status for Kaplan–Meier survival analysis (Figure 3). The median survival time of the sarcopenia + high NLR group was significantly shorter than that of the non-sarcopenia + low NLR group (41 vs. 63 months, Log-rank *P* < 0.001). Similarly, the sarcopenia + high CAR group exhibited a significantly reduced median survival compared to the non-sarcopenia + low CAR group (31 vs. 62 months, Log-rank *P* < 0.001).

## 4 Discussion

This study analyzed a cohort of 318 patients who underwent PD between February 2019 and March 2025 to evaluate the prognostic significance of preoperative sarcopenia, systemic inflammatory status, and their combined impact on postoperative complications and OS. Our findings yielded several key insights. First, preoperative sarcopenia was identified as an independent risk factor for reduced OS in patients following PD. Second, no significant association was observed between sarcopenia and the incidence of postoperative complications. Most importantly, the combination of sarcopenia and a high inflammatory state-specifically elevated NLR and CAR-improved risk-stratification potential for long-term survival compared to the assessment of sarcopenia alone.

Our findings identify sarcopenia as an independent risk factor for OS following PD, a result consistent with a growing body of evidence across various malignancies. For instance, recent studies have established sarcopenia as an independent predictor of poor OS in patients with esophageal cancer undergoing radical radiotherapy (15) and in those with hepatocellular carcinoma following radical resection (16). Specifically concerning pancreatic cancer, a 2024 study corroborated that sarcopenia independently predicts reduced OS after PD (23). Notably, our study builds upon these findings by utilizing a larger sample size and integrating preoperative inflammatory markers into the survival analysis. Our data demonstrate that the combination of sarcopenia with either NLR or CAR provides superior prognostic stratification for OS compared to using these indicators in isolation.

The mechanisms underlying the impact of sarcopenia on OS are multifaceted and complex. Based on a review of existing literature, several key pathways have been identified: First, sarcopenic patients with malignancies often exhibit poor tolerance to mandatory adjuvant therapies, such as chemotherapy, following PD. These individuals are more susceptible to dose reductions or premature treatment discontinuation due to adverse toxicities, which ultimately compromises oncological efficacy (33). Indeed, sarcopenia has been highlighted as a critical driver of adjuvant therapy termination in pancreatic cancer patients post-PD (34). Second, there is a significant “interplay” between sarcopenia and systemic inflammation; when both conditions coexist, patients have an extremely poor prognosis (14). Skeletal muscle cells in sarcopenic states can release pro-inflammatory cytokines, including interleukin-6 (IL-6) and tumor necrosis factor-α (TNF-α) (35). These factors accelerate protein catabolism (36–38), thereby exacerbating muscle wasting and establishing a self-perpetuating “inflammation-sarcopenia” vicious cycle. Third, patients with sarcopenia exhibit reduced secretion of the cytokine interleukin-15 (IL-15) by skeletal muscle cells. Evidence suggests that IL-15 protects natural killer (NK) cells from apoptosis by upregulating Bcl-2 (15). Since NK cells are vital for controlling tumor dissemination, reduced IL-15 levels indirectly impair the host's anti-tumor immune surveillance, leading to tumor cell spread and consequently poor prognosis (39).

Notably, this study found no significant association between sarcopenia or inflammatory markers and postoperative complications evaluated in this study. Our results are consistent with several previous reports. For instance, a retrospective analysis of 162 patients undergoing PD for pancreatic cancer—utilizing three distinct diagnostic criteria—failed to identify sarcopenia as an independent risk factor for postoperative complications (23). Similarly, an Italian study of 371 patients using Martin's criteria (28), reported no significant link between sarcopenic status and adverse postoperative outcomes. However, the literature on this topic remains contentious. In contrast to our findings, a retrospective study of 207 oncological patients undergoing PD demonstrated that sarcopenia was strongly associated with major complications (OR = 8.14; 95% CI: 2.13–43.83; *P* = 0.19) and served as an independent predictor of POPF (OR = 8.50; 95% CI: 2.56–40.18; *P* = 0.008) (26). After reviewing eight studies involving PD for malignancy (19–26), we observed that the relationship between preoperative sarcopenia and postoperative complications following PD remains unclear. This discrepancy may be attributed to several factors, including the lack of standardized SMI cutoffs, heterogeneity across study populations, and the continuous optimization of surgical techniques. Although no significant association was observed in this cohort between CT-defined sarcopenia, immune status and the incidence of postoperative complications following PD, future studies could expand this research framework by examining standardized severity classifications (such as the Clavien-Dindo classification or the ISGPS classification) to provide improved risk stratification for short-term surgical outcomes.

Concurrently, this study found that the combination of sarcopenia with a high inflammatory state (particularly NLR or CAR) further elevates mortality risk. The hazard ratio for the “sarcopenia + high CAR” group reached 2.808 (*P* = 0.001), while that for the “sarcopenia + high NLR” group reached 5.155 (*P* = 0.007). It is worth noting that, although in the subgroup analysis of patients with malignant tumors, sarcopenia itself was no longer a statistically significant independent factor for OS (*P* = 0.203), this may be due to the following reasons: First, the biological characteristics of the tumor itself (e.g., histological type and tumor stage) may mask the impact of sarcopenia on survival. Second, the reduced sample size in the malignant disease subgroup may also be a significant factor influencing the results. However, the combined presence of sarcopenia and a hyperinflammatory state (sarcopenia + high CAR) remains a significant independent risk factor for mortality in cancer patients (HR = 2.193, *P* = 0.037). This suggests that in patients with malignant tumors, cancer may accelerate catabolism by inducing an inflammatory response. This aligns with recent findings that sarcopenia combined with other hyperinflammatory states (such as LMR or PLR) also impacts OS after surgery for other malignancies (e.g., gastric cancer (40), esophageal cancer (41), oral cancer (42), hepatocellular carcinoma (16), and non-metastatic colorectal cancer (13). The underlying mechanism may involve systemic inflammation—a key feature of the tumor microenvironment—activating the ubiquitin-proteasome system and NF-kB signaling pathways through the release of cytokines (e.g., IL-6, TNF-α). This accelerates muscle protein degradation, which in turn locally produces cytokines (17, 18), forming a vicious “inflammation-sarcopenia” cycle. Sarcopenia further weakens immune surveillance, collectively promoting tumor recurrence and metastasis.

This study still has some limitations. First, this was a single-center retrospective study; approximately 11.3% of patients (36 cases) were lost to follow-up, and the inclusion of both benign and malignant diseases in the primary survival analysis introduced selection bias. Secondly, this study established an association between sarcopenia combined with inflammatory markers and OS, but it cannot determine causality. Future large-scale prospective cohort studies or randomized controlled trials are needed to further validate these findings. Thirdly, as this study is retrospective in nature, we were unable to obtain other diagnostic indicators for sarcopenia (such as those assessing muscle strength and physical function) for comparison to validate the findings of this research. Finally, the optimal thresholds for the preoperative inflammatory markers (CAR and NLR) proposed in this paper are reference values derived from a single-center dataset using conventional ROC curves in this study, rather than through time-dependent ROC analysis. Future multicenter prospective studies involving larger external validation cohorts are needed to establish and optimize thresholds with broad applicability.

Nevertheless, our study presents several notable innovations. First, this study innovatively incorporates sarcopenia and inflammatory markers into a comprehensive analysis. Our findings highlight that the coexistence of sarcopenia and a high inflammatory state (elevated NLR or CAR) significantly exacerbates the risk of mortality. Second, The indicators used in this study (based on routine preoperative imaging and complete blood count) can be obtained through standard preoperative examinations without requiring additional costly or complex testing, making them easily applicable across hospitals of all levels. Based on examination results, individualized preoperative intervention strategies can be developed for patients with sarcopenia combined with a pro-inflammatory state (elevated NLR or CAR). Current literature suggests that structured exercise programs combined with nutritional supplementation (such as adequate protein intake, essential amino acids, and vitamin D) may mitigate muscle atrophy (43–46), while anti-inflammatory treatments may modulate the systemic stress response. However, whether such preoperative interventions can delay or even reverse the onset and progression of sarcopenia, alleviate systemic inflammation, and ultimately improve long-term survival rates in patients undergoing PD treatment remains to be further confirmed in future prospective intervention trials.

## 5 Conclusion

In summary, preoperative sarcopenia combined with high NLR or CAR exhibits significant prognostic value for OS following PD. While the findings of this observational study highlight the importance of preoperative nutritional and inflammatory profiling, prospective interventional studies are needed to determine whether targeted strategies to optimize muscle mass and mitigate systemic inflammation can improve long-term survival outcomes in these patients.

## Data Availability

The raw data supporting the conclusions of this article will be made available by the authors, without undue reservation.
